# Impact of pneumococcal conjugate vaccination on pneumococcal nasopharyngeal carriage in the Gambia: Population-based cross-sectional surveys

**DOI:** 10.1016/j.vaccine.2024.02.066

**Published:** 2024-04-11

**Authors:** Grant A Mackenzie, Ilias Hossain, Rasheed Salaudeen, Henry Badji, Ahmed Manjang, Effua Usuf, Christian Bottomley, Brian Greenwood, Philip C Hill

**Affiliations:** a Medical Research Council Unit The Gambia at London School of Hygiene & Tropical Medicine, PO Box 273, Banjul, Gambia; b Murdoch Children’s Research Institute, Parkville, 3052 Melbourne, Victoria, Australia; c Faculty of Infectious and Tropical Diseases, London School of Hygiene & Tropical Medicine, Keppel Street, London WC1E 7HT, UK; d Department of Paediatrics, University of Melbourne, Parkville, 3052 Melbourne, Victoria, Australia; e Tropical Epidemiology Group, London School of Hygiene & Tropical Medicine, Keppel Street, London WC1E 7HT, UK; f Centre for International Health, University of Otago, McMillan Street, Dunedin 9010, New Zealand

**Keywords:** Pneumococcal, Carriage, Pneumococcal conjugate vaccine, Impact, Survey

## Abstract

**Background:**

The introduction of pneumococcal conjugate vaccines (PCV) has reduced carriage of vaccine-type (VT) pneumococci in many settings. We determined the impact of The Gambia’s national PCV programme on carriage of VT pneumococci in the population.

**Methods:**

Seven-valent PCV (PCV7) was introduced in August 2009 without catch-up and with doses scheduled at 2, 3, 4 months of age; it was replaced by PCV13 in May 2011. We did cross-sectional carriage surveys in 2009, 2015, and 2017 in age-stratified, population-based samples. Nasopharyngeal specimens were collected and processed according to WHO guidelines. We calculated observed and adjusted prevalence ratios (PR) of VT carriage before and after PCV introduction.

**Findings:**

We enrolled 2988, 3162, and 2709 participants in 2009, 2015, and 2017 respectively. The baseline (2009) prevalence of VT pneumococcal carriage among children aged 0–4 years was 42.6 %, which declined to 14.9 % and 17.5 % in 2015 and 2017 respectively (adjPR 0.32 [95 % CI 0.27, 0.38] and 0.38 [0.31, 0.46] respectively). VT prevalence among children aged 5–14 years was 16.6 %, 15.1 %, and 15.8 % in the three surveys (2017 vs 2009, adjPR 0.70 [0.58, 0.83]). VT prevalence among 15–44 year-olds was 6.4 %, 5.7 %, and 7.1 % in the three surveys (2017 vs 2009, adjPR 0.59 [0.46, 0.75]), while in those aged ≥ 45 years it was 4.5 %, 6.5 %, and 4.5 % respectively. Non-VT carriage increased in all age-groups. Prevalent residual serotypes were 34 and 15B (age 0–4 years), 3 and 34 (age 5–14 years), and 3 and 16F (age ≥ 15 years).

**Conclusions:**

Introduction of PCV was associated with reduced VT pneumococcal carriage in young, and older children, although with substantial residual prevalence. Persisting VT, and non-VT, carriage indicate significant, persistent transmission of pneumococci in the population.

## Highlights

• Vaccine-type pneumococcal carriage in young and older children declined significantly following routine use of PCV in The Gambia.
• Significant residual vaccine-type carriage in children.
• Non-vaccine-type carriage increased in all age groups.
• Significant pneumococcal transmission continues in the population.

## 1 Introduction

In both high-income [1], [2], [3] and low-income [4], [5], [6] settings, the introduction of pneumococcal conjugate vaccines (PCVs) has been associated with substantial reductions in invasive pneumococcal disease (IPD) caused by vaccine serotypes (VT). Similarly, VT pneumococcal carriage in high-income countries has become very low (i.e. 1 % − 3 %) following the introduction of PCVs [7], [8], [9]. In contrast, significant residual carriage of VT has been observed in young children in Kenyan [6] and Gambian [10] populations which have experienced intense exposure to PCV, as well as in young children in The Gambia and Malawi after 5–6 years of routine PCV use [11], [12].

Similar to reports of serotype replacement in pneumococcal carriage following the introduction of PCV in high-income settings [7], [8], [13], there is increasing evidence of non-vaccine-serotype (NVT) carriage replacement in Africa [6], [10], [12]. Although NVT typically cause less invasive disease [14] the higher rates of transmission of NVT associated with serotype replacement may translate to a higher risk of NVT disease in the population.

The effect of introducing PCV in a high-transmission setting takes time to develop. At least 7 years of PCV use was required in The Gambia for maximal reductions in VT IPD [5]. After 6 years of PCV use in rural Kenya, including extensive catch-up vaccination, VT carriage prevalence continued to fall [6]. The long-term and age-specific effects of routine PCV programmes on pneumococcal carriage are less well documented in sub-Saharan Africa. Carriage studies have been conducted after 5 years of routine PCV use in Malawi [12] and The Gambia [11], and after 4 years in Burkina Faso [15], but only the Burkinabe study included all age groups. Longer term studies including all age groups are important given that serotype replacement in carriage has been mirrored in most settings by replacement IPD over time [1], [3], [16].

We determined the impact of the national PCV programme on pneumococcal carriage in rural Gambia, across all age groups, following 6 and 8 years of routine vaccine use.

## 2 Methods

### 2.1 Setting

The study was conducted in the Basse Health & Demographic Surveillance System (BHDSS) in rural Gambia. The Gambia introduced PCV7 in August 2009, with doses scheduled at 2, 3, and 4 months of age. Children younger than 6 months were eligible to receive all three doses of the vaccine whereas older children were eligible to receive one dose. PCV13 replaced PCV7 in May 2011.

### 2.2 Study design and participants

All participants were residents of the BHDSS, which consisted of 224 villages. The sampling frame was maintained by demographic surveillance consisting of 4-monthly enumerations of all households in the study area.

We conducted cross-sectional surveys of nasopharyngeal carriage in the baseline year of 2009 and in 2015 and 2017, after 6 and 8 years of PCV introduction respectively. We selected 20 villages at random with probability proportional to the number of households in each village. The villages selected in 2009 were also selected in 2015 and 2017. The 2009 survey was designed to measure baseline carriage prevalence in the population and also to investigate VT carriage in 6–10 year-old children who were enrolled as infants in a clinical trial of PCV9 [17], [18]. In 2009, 24 households were randomly selected in each village and all children aged 6–10 years were sampled, while all individuals in other age groups were sampled in every third selected household. The surveys in 2015 and 2017 were designed to compare VT carriage to 2009, in three age strata, 0–4, 5–14, and ≥ 15 years. In these post-PCV surveys, we randomly selected 12 households in each village and used age-stratified lists of household residents to randomly sample equal numbers of individuals by age strata in each household.

### 2.3 Specimen collection and laboratory methods

We recorded demographic information, vaccination history, and carriage risk factors during each survey. Individuals who could not participate were replaced by the next listed household resident in each age group. A calcium alginate swab (Fisher Scientific, Pittsburgh, USA) was collected according to WHO guidelines and then placed it in skimmed-milk-tryptone-glucose-glycerol (STGG) media [19]. Specimens were transported to the laboratory in Basse within 8 h of collection and stored at − 70 °C.

Pneumococci were identified by colony morphology and optochin susceptibility. Colonies with equivocal optochin susceptibility were tested for bile solubility for confirmation as pneumococcal. A single colony, or any morphologically different colonies, were selected from each plate for sub-culture and serotyping. We used latex agglutination [20] to serotype each isolate and when necessary used the Quellung reaction [21] to confirm serotyping. Pneumococcal isolates that failed to react with any of the pooled antisera were classified as non-typeable. Vaccine serotypes were defined as 1, 3, 4, 5, 6A, 6B, 6C, 7F, 9 V, 14, 18C, 19A, 19F, and 23F. Serotype 6C was defined as a VT due to demonstrated vaccine protection associated with PCV13 [3], [22]. We defined VT carriage as carriage of any of the 14 serotypes previously enumerated and NVT carriage as carriage of any other serotype.

### 2.4 Statistical considerations

The 2009 survey aimed to measure population-level carriage prevalence as well as a difference in PCV9 VT carriage in 6–10 year-olds who did and did not receive PCV9 in infancy (E Usuf, PhD Thesis, LSHTM, 2012). The desired sample size among all ages was 2400 [17]. In 2015, a sample size of 1054 in each age strata was chosen, to provide > 99 % power to detect an expected reduction in PCV13 VT prevalence of 75 % in each age group, compared to 2009. In 2017, a sample of 900 in each age group was chosen to provide > 97 % power to detect a 75 % reduction in VT carriage in each age group compared to 2009.

Demographics and other risk factors for carriage were presented for each survey, by age strata. We estimated age-specific prevalence ratios comparing 2015 and 2017 to 2009 by combining data from all participants in the three surveys into a single dataset and fitting a Poisson regression model. Weights were used to account for the oversampling of children aged 6–10 years in the 2009 survey, and robust standard errors were used to adjust p-values and confidence intervals for clustering at the village level [23], [24]. Non-typeable isolates were excluded from regression analyses. We observed an increase in the prevalence of pneumococcal carriage in all age groups in 2015/17 compared to 2009. Therefore, we conducted an adjusted analysis to take account of this secular change in carriage prevalence.

Theoretical and empirical results of VT replacement suggest that PCV introduction does not tend to affect the overall prevalence of pneumococcal carriage [6], [25], [26]. This is because any reduction in VT carriage is compensated for by an increase in NVT carriage. Changes in the overall prevalence of pneumococcal carriage over time may relate to changes in methodology (e.g. quality of NP specimens) or a secular trend that influences pneumococcal carriage (e.g. overall pneumococcal transmission rate). To obtain an estimate for vaccine effectiveness (VE) that accounts for a potential secular change in carriage prevalence, we restricted the Poisson regression analysis to pneumococcal carriers. This analysis provides an unbiased estimate provided that 1) there is complete serotype replacement and 2) any secular change is proportionally the same for VT and NVT. To appreciate why this analysis adjusts for a secular trend, note that these assumptions imply the following model for the relationship between carriage prevalence pre and post PCV:

$$p_{1}^{\mathrm{vt}}=\alpha \left(1-VE\right)p_{0}^{\mathrm{vt}}$$

$$p_{1}^{\mathrm{nvt}}=\alpha \left[p_{0}^{\mathrm{nvt}}+VE\;\times p_{0}^{\mathrm{vt}}\right]$$

where $p_{t}$ = prevalence of carriage at time t (0 = pre PCV, 1 = post PCV) and α = secular change in prevalence. From the model it follows that

$$VE=1-\frac{p_{1}^{\mathrm{vt}}/\left(p_{1}^{\mathrm{vt}}+p_{1}^{\mathrm{nvt}}\right)}{p_{0}^{\mathrm{vt}}/\left(p_{0}^{\mathrm{vt}}+p_{0}^{\mathrm{nvt}}\right)}=1-\frac{post\;PCV\;VT\;prevalence\;among\;carriers}{\;pre\;PCV\;VT\;prevalence\;among\;carriers}$$

Therefore, VE can be estimated by fitting a Poisson regression that is restricted to carriers. We also included a sensitivity analysis in which the Poisson regression included all participants and specified all covariates associated with carriage. VE was calculated as (1 – Prevalence Ratio)*100. Given that changes in NVT and serotype-specific carriage were of secondary importance and that differences between 2015 and 2017 were not of interest, we combined 2015/17 data for these analyses. The level of statistical significance was set at an alpha level of 0.05. All statistical analyses were done using R version 4.2.1.

### 2.5 Ethical considerations

Ethical approval for the study was granted by the Gambia Government/MRCG Joint Ethics Committee (numbers 1148, 1418, and 1542). Written informed consent was obtained from participants aged ≥ 15 years, from the parent or guardian of participants aged 0–14 years, and assent was obtained from youth aged 12–14 years.

## 3 Results

### 3.1 Enrolment and participant characteristics

In 2009, 2015, and 2017 we enrolled 2988, 3162, and 2709 participants respectively (Table 1). As expected, the proportion of participants in the 5–14-year age group was greater in 2009 given the over-sampling of 6–10-year-olds that year [17]. Among participants aged 0–4 years, the proportion who had received two or more doses of PCV was 86 % (875/1017) in 2015 and 73 % (672/919) in 2017.

**Table 1:** Epidemiological characteristics of participants.

Characteristic		2009	2015	2017	p-value
		N = 2988 n (%)	N = 3162 n (%)	N = 2709 n (%)	
Age (years)	0–4	530 (17.7)	1017 (32.2)	919 (33.9)	0.01
	5–14	1424 (47.7)	1083 (34.3)	800 (29.5)	
	15–44	791 (26.5)	802 (25.4)	748 (27.6)	
	≥45	243 (8.1)	260 (8.2)	242 (8.9)	
Sex	Male	1386 (46.4)	1407 (44.5)	1192 (44.0)	0.33
Ethnicity	Mandinka	723 (24.2)	677 (21.4)	591 (21.8)	0.21
	Fula	648 (21.7)	860 (27.2)	555 (20.5)	
	Serahule	1596 (53.4)	1622 (51.3)	1555 (57.4)	
	Other	20 (0.7)	3 (0.1)	8 (0.3)	
Season	Dry	875 (29.3)	1173 (37.1)	672 (24.8)	0.53
	Wet	2113 (70.7)	1989 (62.9)	2037 (75.2)	
Doses of PCV in 0–4 years	0–1	529 (100)	143 (14.0)	246 (26.8)	<0.001
	2–3	0 (0)	875 (86.0)	672 (73.2)	
No. children 0–4 years in household	0–4	960 (32.1)	860 (27.2)	867 (32.0)	0.74
	5–9	1138 (38.1)	1328 (42.0)	1124 (41.5)	
	≥10	890 (29.8)	974 (30.7)	718 (26.5)	
Household size	0–19	630 (21.1)	617 (19.5)	471 (17.4)	0.48
	20–49	1736 (58.1)	1685 (53.3)	1582 (58.4)	
	≥50	622 (20.8)	860 (27.2)	656 (24.2)	
Bed sharing	Yes	1753 (58.8)	1806 (57.2)	1274 (47.0)	<0.001
Cooking method	Firewood	2952 (98.8)	3146 (99.5)	2709 (100)	0.56
	Charcoal/ Other	36 (1.1)	16 (0.5)	0 (0)	
Cooking place	Inside	2229 (74.6)	3062 (96.9)	2644 (97.6)	0.08
	Outside	84 (2.8)	60 (1.9)	3 (0.1)	
	Inside & outside	675 (22.6)	38 (1.2)	60 (2.2)	

Missing values –	2009	2015	2017
Ethnicity	1	1	10
No. children 0–4 years in household	167	0	1
Household size	167	0	1
Cooking method	184	0	25
Cooking place	181	0	0

P-values calculated using a chi-square test adjusted for village-level clustering.

### 3.2 Pneumococcal carriage

**Fig. 1:**
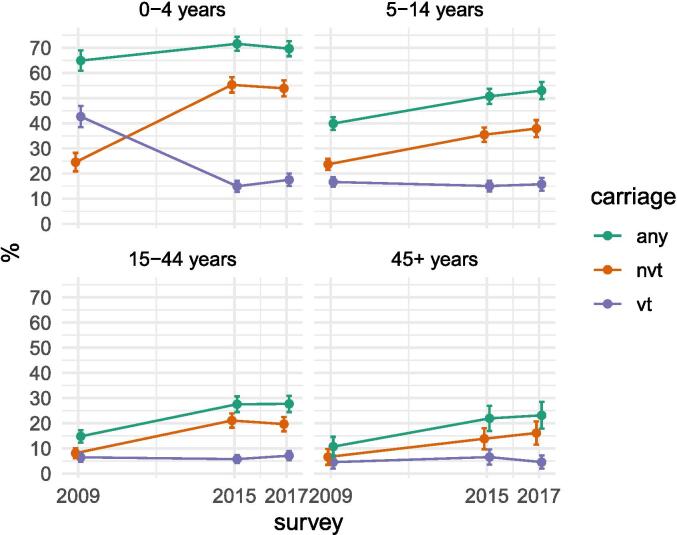
Age-specific prevalence of vaccine-type and non-vaccine-type pneumococci in 2009, 2015, and 2017.

Among all age groups, the proportion of participants carrying any pneumococci in 2015/17 increased compared to 2009, and this increase was most pronounced in the older age groups Fig. 1. Non-typeable pneumococcal isolates were uncommon (∼1.0 – 1.5 %) and of similar prevalence in each survey (Supplementary table 1). Risk factors for pneumococcal carriage are shown in Supplementary table 1.

### 3.3 Vaccine-type pneumococcal carriage

Table 2 reports carriage prevalence at baseline in 2009 and in 2015 and 2017 Fig. 1, with observed and adjusted VT prevalence ratios. Prevalence ratios from the sensitivity analysis including all participants and specifying all covariates associated with carriage (Supplementary table 2) were similar to the observed prevalence ratios (Table 2). Adjusted and observed vaccine effectiveness estimates differed by > 10 % in the older child and adult age groups and by 8 % in the 0–4-year age group. Thus, a significant confounding effect is evident, related to the effect which increased pneumococcal carriage in 2015/17 compared to 2009. Therefore, the VE estimates based on the adjusted prevalence ratios are the most valid (Table 2).

**Table 2:** Age-specific pneumococcal carriage prevalence in each survey and vaccine-type prevalence ratios comparing 2015 and 2017 to 2009.

			Survey year			Observ. prevalence ratio 2015 v 2009 (95 % CI)	Adj. prevalence ratio 2015 v 2009 (95 % CI)	Observ. prevalence ratio 2017 v 2009 (95 % CI)	Adj. prevalence ratio 2017 v 2009 (95 % CI)
			2009 n (%)	2015 n (%)	2017 n (%)				
Age group (in years)	0–4		N = 530	N = 1017	N = 919				
		VT	226 (42.6)	152 (14.9)	161 (17.5)	0.35 (0.27, 0.45)	0.32 (0.27, 0.38)	0.41 (0.32, 0.53)	0.38 (0.31, 0.46)
		NVT	130 (24.5)	562 (55.3)	495 (53.9)				
		NT	8 (1.5)	16 (1.6)	10 (1.1)				
		*Spn	344 (64.9)	728 (71.6)	640 (69.6)				
	5–14		N = 1424	N = 1083	N = 800				
		VT	237 (16.6)	163 (15.1)	126 (15.8)	0.83 (0.69, 0.99)	0.65 (0.55, 0.76)	0.94 (0.71, 1.23)	0.70 (0.58, 0.83)
		NVT	337 (23.7)	384 (35.5)	303 (37.9)				
		NT	20 (1.4)	18 (1.7)	13 (1.6)				
		*Spn	568 (39.9)	549 (50.7)	424 (53.0)				
	15–44		N = 791	N = 802	N = 748				
		VT	51 (6.4)	46 (5.7)	53 (7.1)	0.89 (0.53, 1.51)	0.47 (0.35, 0.64)	1.10 (0.73, 1.66)	0.59 (0.46, 0.75)
		NVT	64 (8.1)	169 (21.1)	147 (19.7)				
		NT	5 (0.6)	7 (0.9)	10 (1.3)				
		*Spn	117 (14.8)	221 (27.6)	207 (27.7)				
	≥45		N = 243	N = 260	N = 242				
		VT	11 (4.5)	17 (6.5)	11 (4.5)	1.44 (0.75, 2.76)	0.76 (0.37, 1.53)	1.00 (0.48, 2.1)	0.53 (0.25, 1.14)
		NVT	16 (6.6)	36 (13.8)	39 (16.1)				
		NT	0 (0.0)	4 (1.5)	7 (2.9)				
		*Spn	26 (10.7)	57 (21.9)	56 (23.1)				

Observed (Observ.) prevalence ratios obtained from a weighted Poisson regression without covariates and including all participants, i.e. not adjusted for a trend in pneumococcal carriage. Adjusted (Adj.) prevalence ratios obtained from the same regression but restricted to pneumococcal carriers, i.e. adjusted for a trend in pneumococcal carriage. VT: Vaccine-type, NVT: non-vaccine type, NT: non-typeable, Spn: *S. pneumoniae*. *Any Spn, i.e. VT or NVT or NT.

In the 0–4-year age group, the adjusted estimates of VE in 2015 and 2017 were 68 % (95 % CI; 62 %, 73 %) and 62 % (54 %, 69 %) respectively (Table 2). Thus, the PCV programme was associated with an estimated reduction in VT carriage of 62 %-68 % in this age group. The PCV programme was associated with an estimated 30 %-35 % reduction in VT carriage in the 5–14-year age group. In the 15–44-year age group, the PCV programme was associated with an estimated 41 %-53 % reduction in VT carriage. In the ≥ 45-year age group, the adjusted estimates of VE in 2015 and 2017 were 24 % and 47 % respectively, although wide confidence intervals indicate substantial uncertainty regarding the effect of the PCV programme to reduce VT carriage in this age group (Table 2).

In all age groups, the estimates of VE did not differ significantly when comparing 2015 and 2017 (Table 2). Thus, the effect of the PCV programme on VT carriage appeared to have developed fully within 6 years.

### 3.4 Non-vaccine-type pneumococcal carriage

The prevalence of NVT carriage increased substantially between 2009 and 2015/17 in all age groups (Table 2). Prevalence estimates in 2015 and 2017 were more than double those in 2009 in the 0–4-year, 15–44-year, and ≥ 45 year age groups.

### 3.5 Serotype-specific pneumococcal carriage

**Fig. 2:**
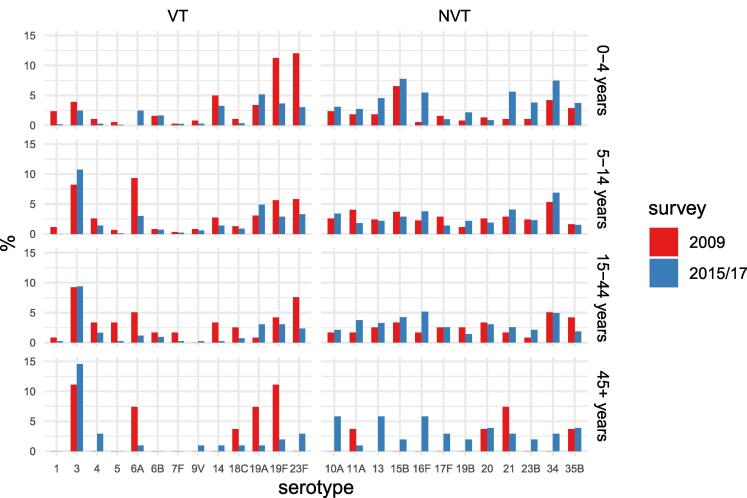
Age-specific prevalence of serotype-specific pneumococcal carriage in 2009 and 2015/17.

Detailed results of serotype-specific carriage in 2009 compared to 2015/17 are shown in Supplementary table 3. Comparing 2015/17 to 2009 in the 0–4-year age group, there were significant reductions in the prevalence of serotypes 19F (11 % to 3 %) and 23F (12 % to 3 %) [Fig. 2]. Persisting VT pneumococci in 2015/2017 in this age group were 19A (6 %), 14 (3 %), 19F (3 %), and 23F (3 %). The most prevalent serotypes were 34 (8 %) and 15B (8 %). In the 5–14-year age group, reduced prevalence in 2015/17 was observed for serotypes 6A (11 % to 3 %) and 19F (6 % to 3 %) and persisting VT carriage comprised mainly types 3 (11 %) and 19A (5 %). The most prevalent serotypes in 2015/17 were 3 (11 %) and 34 (7 %). In the 15–44-year age group the prevalence of serotypes 6A (5 % to 1 %) and 23F (8 % to 2 %) declined and serotype 3 (9 %) was the dominant persisting VT with smaller proportions of 19F (3 %) and 19A (3 %). Serotypes 16F (5 %) and 34 (5 %) were the most prevalent in 2015/17. In those aged ≥ 45 years serotype 6A (7 % to 1 %) declined in 2015/17 and the main persisting VT was 3 (14 %) with smaller proportions of 4 (3 %) and 23F (3 %). Other prevalent serotypes were 13 (6 %), 16F (6 %), and 10A (6 %). The prevalence of serotype 3 was similar in the baseline and post-PCV surveys.

The prevalence of some, but not all, NVT increased in 2015/2017 (Fig. 2). NVT present at baseline that increased significantly were 13, 19B, 21, 23B, and 16F, while 15B, 35B, 11A, 10A, and 20 were stable. In the age group ≥ 45 years, several serotypes undetected at baseline were found prevalent in 2015/17, namely types 13, 34, 16F, and 10A.

## 4 Discussion

In this population-based study in rural Gambia, following 8 years of routine infant vaccination with PCV, there was a substantial reduction in VT carriage in young children. We also observed a reduction in VT carriage in the 5–14-year age group, although of lesser magnitude. Reductions in VT carriage in 2015 and 2017 compared to 2009 did not differ, indicating that the full effect of the PCV programme had developed after 6 years. After 8 years of PCV introduction, significant residual carriage of VT pneumococci remained in all age groups. The prevalence of NVT increased substantially with an expanded number of circulating NVT serotypes.

The 60 %-65 % reduction in VT carriage in the 0–4 year age group is similar to the reductions seen 3 years after PCV10 introduction in Mozambique [27] and after 4 years of PCV13 in South Africa [28]. The 74 % reduction in VT carriage in the 0–4-year age group observed in Kenya 6 years after the introduction of PCV10 [6] and the 76 %-80 % reduction in rural western Gambia after 10 years of PCV pressure [10] appear greater than what we observed. The greater impact seen in these two studies may have been due to the greater vaccine pressure of catch-up vaccination to 5 years of age in Kenya and an earlier mass PCV vaccination study in the western Gambia site. On the other hand, the impact on VT that we observed seems greater than the 20 %-40 % decline observed 5 years after the introduction of PCV13 in Malawi [12]. Of note, baseline VT prevalence in young children in Malawi was around 20 %, in contrast to 34 % in Kenya [6] and over 40 % in The Gambia [10], South Africa [28], and Mozambique [27]. It is unclear why PCV13 impact on VT carriage in Malawi may be less than in other high-transmission settings; the investigators in Malawi have correlated the effect with a high ‘force of infection’ in a mathematical model [29] and observed waning of population immunity late in the 1st year and in the 2nd year of life following the PCV schedule including three early infant doses [30]. Unlike low-transmission settings [31], the impact of PCV introduction on VT carriage in high-transmission settings may vary with the intensity and duration of vaccine pressure as well as factors that influence transmission of pneumococci in the population.

The effect of the PCV programme on VT carriage varied by age group with lesser impact in older children, who primarily experienced only the indirect effects of vaccination. Similar indirect herd effects have been seen among older children in The Gambia [10], Malawi [12], and Kenya [6].

We observed a substantial increase in pneumococcal carriage in 2015/17 compared to 2009 among adults and older children (11 % − 13 % absolute increase) and to a lesser extent in younger children (5 % − 7 % absolute increase). Different field and laboratory staff conducted the 2009 and 2015/17 surveys. Despite no change in standard operating procedures changes in the technique and quality of NP specimens or culture practice may explain the increase in pneumococcal carriage. The increase in overall pneumococcal carriage may be explained by an increase in rates of transmission in the population, manifesting to a greater degree in older age groups with lower baseline prevalence. An increase in the duration of carriage may also lead to increased prevalence, however the prevalent residual serotypes, 3, 16F, and 34, are characterised by only moderate durations of carriage [32]. The prevalence of non-typeable pneumococci was very low (1 %-2% in each survey year), although prevalence in the ≥ 45 years age group was 0 %, 1.5 %, and 2.9 % in the three surveys. The potential explanations for increased pneumococcal carriage are very unlikely to have differential effects for VT and NVT. Thus, the presumed secular effect increasing pneumococcal carriage is likely to be proportionally the same for VT and NVT, although this cannot be proved. The adjusted results from the analysis restricted to pneumococcal carriers are plausible but should be interpreted with caution. They are consistent with the indirect herd effect of PCV programmes on VT carriage among adults elsewhere in The Gambia [11], Kenya [6], and South Africa [28]. The observed prevalence ratio results are difficult to interpret given the secular effect of increased pneumococcal carriage in 2015/17.

The persistent VT carriage that we observed is consistent with reports from other high-transmission settings. Our observed 18 % VT prevalence in the 0–4 year age group in 2017 is similar to the 14 % in the Gambian site that experienced past mass vaccination [10], 11 % in an urban Gambian location [11], 16 % in Malawi [12], and 15 % in Mozambique [27]. This significant residual prevalence of VT carriage indicates ongoing transmission in the community and calls for the evaluation of alternative approaches to the use of PCV. We observed 15 % VT prevalence in children aged 5–14 years in 2017, similar to that in young children, indicating that older children are likely to comprise a significant reservoir of VT transmission. Similar observations have been made in older children elsewhere in The Gambia [10], Malawi [12], and Kenya [6]. Modelling studies that account for local interpersonal contact patterns may help predict the outcome of different approaches to vaccination of older age groups and the use of booster doses. Empiric studies are also needed to evaluate the effect of vaccinating older groups, booster doses at different ages, differing intervals between doses, and the role of one- or two-stage vaccination campaigns. The earlier trial of mass PCV7 vaccination in Gambian villages did not interrupt VT transmission and the effects in different age groups varied with time [33]. It is likely that more potent vaccines will be needed in high-transmission settings.

Despite significant VT transmission, we see very low incidence of VT IPD among young children in the BHDSS [5]. Vaccine-induced immunity appears sufficient to prevent IPD in the target age group while reducing but not eliminating carriage acquisition. Surveillance does not, however, detect potential ongoing cases of non-invasive VT disease, such as pneumonia, against which the efficacy of PCV may be less than against IPD [34]. The increase in NVT carriage highlights the potential for serotype replacement disease, which has been consistently observed in high-income settings, particularly in susceptible populations with co-morbidities and the elderly [3]. Surveillance to date in high-transmission settings indicates relatively small absolute increases in NVT disease incidence [4], [6]. High quality surveillance for IPD, pneumonia, and carriage in several African sites will be needed to determine the time course of the unfolding impact of PCV programmes.

The strengths of our study include population-based random sampling of all ages, substantial sample sizes, and high-quality pneumococcal serotyping with only 1 % of isolates unable to be serotyped. However, there are limitations. Several risk factor variables were omitted in 2009 and could not be compared across all surveys. The increased detection of pneumococcal carriage in 2015/2017 compared to 2009, could have been due to an unidentified change in study methodology or a secular trend in pneumococcal transmission. The results of the adjusted analysis need to be interpreted with caution given the assumptions of the underlying model and the unexplained increase in pneumococcal carriage in 2015/17.

A research priority to address serotype replacement is the development of higher valency vaccines. Population-based surveillance for IPD and pneumonia are lacking in most low-income countries. Funding agencies that have facilitated the uptake of PCV in low-income countries should recognise their ongoing role to support monitoring of the impact of PCV interventions.

Our findings in rural Gambia show that following 8 years of the introduction of routine infant vaccination with PCV, there was a substantial reduction in VT carriage in young children. However, significant residual VT carriage remains in all age groups. The prevalence of NVT increased substantially with an expanded number of NVT serotypes, representing ongoing risk for serotype replacement disease.

## CRediT authorship contribution statement

**Grant A Mackenzie:** Conceptualization, Data curation, Funding acquisition, Methodology, Project administration, Resources, Supervision, Writing – original draft, Writing – review & editing. **Ilias Hossain:** Data curation, Investigation, Methodology, Project administration, Supervision, Validation, Writing – review & editing. **Rasheed Salaudeen:** Data curation, Investigation, Methodology, Project administration, Supervision, Validation, Writing – review & editing. **Henry Badji:** Investigation, Methodology, Project administration, Supervision, Validation, Writing – review & editing. **Ahmed Manjang:** Data curation, Investigation, Methodology, Project administration, Supervision, Writing – review & editing. **Effua Usuf:** Conceptualization, Data curation, Investigation, Methodology, Project administration, Supervision, Validation, Writing – review & editing. **Christian Bottomley:** Conceptualization, Formal analysis, Validation, Visualization, Writing – original draft, Writing – review & editing. **Brian Greenwood:** Conceptualization, Formal analysis, Writing – original draft, Writing – review & editing. **Philip C Hill:** Conceptualization, Formal analysis, Funding acquisition, Investigation, Methodology, Resources, Writing – original draft, Writing – review & editing.

## Declaration of competing interest

The authors declare that they have no known competing financial interests or personal relationships that could have appeared to influence the work reported in this paper.

## Data Availability

Data will be made available on request.
