# Supplementary material for: Impact of pneumococcal conjugate vaccination on pneumococcal nasopharyngeal carriage in the Gambia: Population-based cross-sectional surveys
Source: Vaccine. 2024 Apr 11;42(10):2680–6. doi: 10.1016/j.vaccine.2024.02.066 (PMC11004668; doi:10.1016/j.vaccine.2024.02.066)
Supplement: Supplementary data 1 [file mmc1.docx]

**Impact of pneumococcal conjugate vaccination on pneumococcal nasopharyngeal carriage in The Gambia: population-based cross-sectional surveys**

**Online supplementary material**

**Supplementary Table 1.** Analysis of risk factors for pneumococcal carriage

|  | N | n | % | Odds Ratio | 95% CI | p-value | Adj. Odds Ratio | 95% CI | p-value |
| --- | --- | --- | --- | --- | --- | --- | --- | --- | --- |
| Age |  |  |  |  |  | <0.001 |  |  | <0.001 |
| 0-4 years | 2466 | 1712 | 69.42 |  |  |  |  |  |  |
| 5-14 years | 3307 | 1541 | 46.6 | 0.38 | 0.34-0.42 |  | 0.43 | 0.39-0.48 |  |
| 15-44 years | 2341 | 545 | 23.28 | 0.13 | 0.11-0.16 |  | 0.14 | 0.12-0.17 |  |
| 45+ years | 745 | 139 | 18.66 | 0.1 | 0.08-0.13 |  | 0.11 | 0.09-0.14 |  |
| Sex |  |  |  |  |  | <0.001 |  |  | 0.03 |
| Male | 3984 | 1975 | 49.57 |  |  |  |  |  |  |
| Female | 4875 | 1962 | 40.25 | 0.69 | 0.63-0.76 |  | 0.88 | 0.78-0.99 |  |
| Ethnicity |  |  |  |  |  | 0.06 |  |  | 0.13 |
| Mandinka | 1986 | 889 | 44.76 |  |  |  |  |  |  |
| Fula | 2062 | 973 | 47.19 | 1.1 | 0.88-1.38 |  | 1.08 | 0.85-1.38 |  |
| Sarahule | 4766 | 2057 | 43.16 | 0.94 | 0.84-1.06 |  | 0.89 | 0.80-1.00 |  |
| Other | 33 | 10 | 30.3 | 0.54 | 0.30-0.96 |  | 0.71 | 0.34-1.46 |  |
| Season |  |  |  |  |  | 0.26 |  |  | 0.62 |
| Dry | 2720 | 1297 | 47.68 |  |  |  |  |  |  |
| Rainy | 6139 | 2640 | 43 | 0.83 | 0.60-1.15 |  | 0.93 | 0.69-1.26 |  |
| No. children <5 years in household |  |  |  |  |  | 0.14 |  |  | 0.95 |
| 0-4 | 2633 | 1114 | 42.31 |  |  |  |  |  |  |
| 5-9 | 3528 | 1619 | 45.89 | 1.16 | 0.98-1.37 |  | 1.02 | 0.87-1.19 |  |
| 10+ | 2530 | 1158 | 45.77 | 1.15 | 0.96-1.38 |  | 1.02 | 0.87-1.19 |  |
| Household size |  |  |  |  |  | 0.25 |  |  | 0.39 |
| 0-19 | 1682 | 724 | 43.04 |  |  |  |  |  |  |
| 20-49 | 4907 | 2217 | 45.18 | 1.09 | 0.98-1.21 |  | 1.08 | 0.95-1.23 |  |
| 50+ | 2102 | 950 | 45.2 | 1.09 | 0.90-1.32 |  | 1.01 | 0.86-1.19 |  |
| Cooking method |  |  |  |  |  | <0.001 |  |  | <0.001 |
| Firewood | 8601 | 3846 | 44.72 |  |  |  |  |  |  |
| Charcoal | 23 | 15 | 65.22 | 2.32 | 1.60-3.37 |  | 1.65 | 1.30-2.10 |  |
| Other | 26 | 7 | 26.92 | 0.46 | 0.22-0.96 |  | 0.66 | 0.45-0.97 |  |
| Cooking place |  |  |  |  |  | <0.001 |  |  | 0.05 |
| Inside kitchen | 7803 | 3554 | 45.55 |  |  |  |  |  |  |
| Outside kitchen | 51 | 20 | 39.22 | 0.77 | 0.48-1.24 |  | 1.08 | 0.75-1.55 |  |
| Both inside and outside | 732 | 264 | 36.07 | 0.67 | 0.54-0.84 |  | 0.91 | 0.72-1.15 |  |
| Outside in the open | 92 | 49 | 53.26 | 1.36 | 1.09-1.70 |  | 1.26 | 1.05-1.51 |  |

Adj. odds ratio: odds ratio adjusted for age, sex, and survey year. 95% confidence intervals adjusted for village-level clustering.

**Supplementary Table 2.** Sensitivity analysis - pneumococcal carriage in each survey and vaccine-type prevalence ratios comparing 2015 and 2017 to 2009

|  | | | Survey year | | | Sensitivity analysis adj.  prevalence ratio 2015 v  2009 (95% CI) | Sensitivity analysis adj.  prevalence ratio 2017 v 2009 (95% CI) |
| --- | --- | --- | --- | --- | --- | --- | --- |
|  |  |  | 2009  n (%) | 2015  n (%) | 2017  n (%) |  |  |
| Age group  (in years) | 0-4 |  | N=530 | N=1017 | N=919 |  |  |
|  |  | VT | 226 (42.6) | 152 (14.9) | 161 (17.5) | 0.36 (0.28, 0.46) | 0.43 (0.33, 0.55) |
|  |  | NVT | 130 (24.5) | 562 (55.3) | 495 (53.9) |  |  |
|  |  | NT | 8 (1.5) | 16 (1.6) | 10 (1.1) |  |  |
|  |  | Spn | 344 (64.9) | 728 (71.6) | 640 (69.6) |  |  |
|  | 5-14 |  | N=1424 | N=1083 | N=800 |  |  |
|  |  | VT | 237 (16.6) | 163 (15.1) | 126 (15.8) | 0.78 (0.62, 0.97) | 0.92 (0.69, 1.23) |
|  |  | NVT | 337 (23.7) | 384 (35.5) | 303 (37.9) |  |  |
|  |  | NT | 20 (1.4) | 18 (1.7) | 13 (1.6) |  |  |
|  |  | Spn | 568 (39.9) | 549 (50.7) | 424 (53.0) |  |  |
|  | 15-44 |  | N=791 | N=802 | N=748 |  |  |
|  |  | VT | 51 (6.4) | 46 (5.7) | 53 (7.1) | 0.71 (0.46, 1.10) | 0.93 (0.63, 1.37) |
|  |  | NVT | 64 (8.1) | 169 (21.1) | 147 (19.7) |  |  |
|  |  | NT | 5 (0.6) | 7 (0.9) | 10 (1.3) |  |  |
|  |  | Spn | 117 (14.8) | 221 (27.6) | 207 (27.7) |  |  |
|  | ≥45 |  | N=243 | N=260 | N=242 |  |  |
|  |  | VT | 11 (4.5) | 17 (6.5) | 11 (4.5) | 1.45 (0.75, 2.80) | 0.96 (0.47, 1.97) |
|  |  | NVT | 16 (6.6) | 36 (13.8) | 39 (16.1) |  |  |
|  |  | NT | 0 (0.0) | 4 (1.5) | 7 (2.9) |  |  |
|  |  | Spn | 26 (10.7) | 57 (21.9) | 56 (23.1) |  |  |

Sensitivity analysis adjusted (adj.) prevalence ratios from a weighted Poisson regression including all participants and adjusted for season, age, no. household children <5, household size, cooking place, and village, i.e. not adjusted for a secular trend in pneumococcal carriage. VT: Vaccine-type, NVT: non-vaccine type, NT: non-typeable, Spn: *S. pneumoniae*.

**Supplementary Table 3.** Prevalence of serotype-specific pneumococcal carriage in 2009 and 2015/17, by age group

|  | **Age 0-4 years** | | | | | | | | | **Age 5-14 years** | | | | | | | | **Age 15-44 years** | | | | | | | | **Age 45+ years** | | | | | | | |
| --- | --- | --- | --- | --- | --- | --- | --- | --- | --- | --- | --- | --- | --- | --- | --- | --- | --- | --- | --- | --- | --- | --- | --- | --- | --- | --- | --- | --- | --- | --- | --- | --- | --- |
| **Sero**  **type** | **2009** | | | **2015/17** | | | |  | | **2009** | | | **2015/17** | | |  | | | **2009** | | | **2015/17** | | |  | | **2009** | | **2015/17** | |  | | |
|  | **n/N** | **%** | | **n/N** | | **%** | | **p-**v**alue** | | **n/N** | | **%** | | **n/N** | **%** | **p-value** | | | **n/N** | **%** | **n/N** | | **%** | **p-value** | | | **n/N** | **%** | **n/N** | **%** | | **p-value** | |
| **VT** |  |  | |  | |  | |  | |  | |  | |  |  |  | | |  |  |  | |  |  | | |  |  |  |  | |  | |
| 1 | 9/383 | 2.3 | | 2/1395 | | 0.1 | | 0.074 | | 7/621 | | 1.1 | | 0/1005 | 0 | 0.033 | | | 1/119 | 0.8 | 1/426 | | 0.2 | 0.696 | | | 0/27 | 0 | 0/103 | 0 | | NA | |
| 3 | 15/383 | 3.9 | | 34/1395 | | 2.4 | | 0.37 | | 51/621 | | 8.2 | | 108/1005 | 10.7 | 0.042 | | | 11/119 | 9.2 | 40/426 | | 9.4 | 0.075 | | | 3/27 | 11.1 | 15/103 | 14.6 | | 0.094 | |
| 4 | 4/383 | 1 | | 4/1395 | | 0.3 | | 0.236 | | 16/621 | | 2.6 | | 14/1005 | 1.4 | 0.124 | | | 4/119 | 3.4 | 7/426 | | 1.6 | 0.567 | | | 0/27 | 0 | 3/103 | 2.9 | | 0.104 | |
| 5 | 2/383 | 0.5 | | 1/1395 | | 0.1 | | 0.427 | | 4/621 | | 0.6 | | 1/1005 | 0.1 | 0.508 | | | 4/119 | 3.4 | 1/426 | | 0.2 | 0.175 | | | 0/27 | 0 | 0/103 | 0 | | NA | |
| 6A | 82/383 | 21.4 | | 34/1395 | | 2.4 | | 0.002 | | 58/621 | | 9.3 | | 30/1005 | 3 | 0.003 | | | 6/119 | 5 | 5/426 | | 1.2 | 0.218 | | | 2/27 | 7.4 | 1/103 | 1 | | 0.21 | |
| 6B | 6/383 | 1.6 | | 23/1395 | | 1.6 | | 0.858 | | 5/621 | | 0.8 | | 7/1005 | 0.7 | 0.659 | | | 2/119 | 1.7 | 4/426 | | 0.9 | 0.409 | | | 0/27 | 0 | 0/103 | 0 | | NA | |
| 7F | 1/383 | 0.3 | | 3/1395 | | 0.2 | | 0.925 | | 2/621 | | 0.3 | | 2/1005 | 0.2 | 0.967 | | | 2/119 | 1.7 | 1/426 | | 0.2 | 0.328 | | | 0/27 | 0 | 0/103 | 0 | | NA | |
| 9V | 3/383 | 0.8 | | 4/1395 | | 0.3 | | 0.424 | | 5/621 | | 0.8 | | 6/1005 | 0.6 | 0.556 | | | 0/119 | 0 | 1/426 | | 0.2 | 0.317 | | | 0/27 | 0 | 1/103 | 1 | | NA | |
| 14 | 19/383 | 5 | | 45/1395 | | 3.2 | | 0.17 | | 17/621 | | 2.7 | | 14/1005 | 1.4 | 0.207 | | | 4/119 | 3.4 | 1/426 | | 0.2 | 0.277 | | | 0/27 | 0 | 1/103 | 1 | | NA | |
| 18C | 4/383 | 1 | | 5/1395 | | 0.4 | | 0.302 | | 8/621 | | 1.3 | | 9/1005 | 0.9 | 0.46 | | | 3/119 | 2.5 | 3/426 | | 0.7 | 0.819 | | | 1/27 | 3.7 | 1/103 | 1 | | NA | |
| 19A | 13/383 | 3.4 | | 72/1395 | | 5.2 | | 0.068 | | 19/621 | | 3.1 | | 49/1005 | 4.9 | 0.022 | | | 1/119 | 0.8 | 13/426 | | 3.1 | 0.08 | | | 2/27 | 7.4 | 1/103 | 1 | | 0.248 | |
| 19F | 43/383 | 11.2 | | 51/1395 | | 3.7 | | 0.004 | | 35/621 | | 5.6 | | 29/1005 | 2.9 | 0.114 | | | 5/119 | 4.2 | 13/426 | | 3.1 | 0.858 | | | 3/27 | 11.1 | 2/103 | 1.9 | | 0.541 | |
| 23F | 46/383 | 12 | | 42/1395 | | 3 | | 0.013 | | 36/621 | | 5.8 | | 33/1005 | 3.3 | 0.301 | | | 9/119 | 7.6 | 10/426 | | 2.3 | 0.399 | | | 0/27 | 0 | 3/103 | 2.9 | | 0.127 | |
| **NVT** |  |  | |  | |  | |  | |  | |  | |  |  |  | | |  |  |  | |  |  | | |  |  |  |  | | c | |
| 10A | 9/383 | 2.3 | | 43/1395 | | 3.1 | | 0.275 | | 16/621 | | 2.6 | | 34/1005 | 3.4 | 0.128 | | | 2/119 | 1.7 | 9/426 | | 2.1 | 0.208 | | | 0/27 | 0 | 6/103 | 5.8 | | 0.05 | |
| 11A | 7/383 | 1.8 | | 38/1395 | | 2.7 | | 0.875 | | 25/621 | | 4 | | 18/1005 | 1.8 | 0.339 | | | 2/119 | 1.7 | 16/426 | | 3.8 | 0.044 | | | 1/27 | 3.7 | 1/103 | 1 | | NA | |
| 13 | 7/383 | 1.8 | | 63/1395 | | 4.5 | | 0.009 | | 15/621 | | 2.4 | | 22/1005 | 2.2 | 0.909 | | | 3/119 | 2.5 | 14/426 | | 3.3 | 0.284 | | | 5/27 | 18.5 | 6/103 | 5.8 | | 0.857 | |
| 15B | 25/383 | 6.5 | | 108/1395 | | 7.7 | | 0.385 | | 23/621 | | 3.7 | | 29/1005 | 2.9 | 0.459 | | | 4/119 | 3.4 | 18/426 | | 4.2 | 0.13 | | | 0/27 | 0 | 2/103 | 1.9 | | NA | |
| 16F | 2/383 | 0.5 | | 76/1395 | | 5.4 | | 0.001 | | 14/621 | | 2.3 | | 38/1005 | 3.8 | 0.228 | | | 2/119 | 1.7 | 22/426 | | 5.2 | 0.054 | | | 0/27 | 0 | 6/103 | 5.8 | | 0.135 | |
| 17F | 6/383 | 1.6 | | 14/1395 | | 1 | | 0.437 | | 18/621 | | 2.9 | | 14/1005 | 1.4 | 0.213 | | | 3/119 | 2.5 | 11/426 | | 2.6 | 0.388 | | | 0/27 | 0 | 3/103 | 2.9 | | 0.085 | |
| 19B | 3/383 | 0.8 | | 30/1395 | | 2.2 | | 0.012 | | 7/621 | | 1.1 | | 22/1005 | 2.2 | 0.368 | | | 3/119 | 2.5 | 6/426 | | 1.4 | 0.876 | | | 0/27 | 0 | 2/103 | 1.9 | | NA | |
| 20 | 5/383 | 1.3 | | 12/1395 | | 0.9 | | 0.163 | | 16/621 | | 2.6 | | 19/1005 | 1.9 | 0.586 | | | 4/119 | 3.4 | 13/426 | | 3.1 | 0.179 | | | 1/27 | 3.7 | 4/103 | 3.9 | | 0.419 | |
| 21 | 4/383 | | 1 | | 78/1395 | | 5.6 | | 0.002 | | 18/621 | 2.9 | | 41/1005 | 4.1 | 0.06 | | | 2/119 | 1.7 | 11/426 | | 2.6 | 0.126 | | | 2/27 | 7.4 | 3/103 | 2.9 | | 0.758 | |
| 23B | 4/383 | | 1 | | 53/1395 | | 3.8 | | 0.009 | | 15/621 | 2.4 | | 23/1005 | 2.3 | 0.828 | | | 1/119 | 0.8 | 9/426 | | 2.1 | 0.048 | | | 0/27 | 0 | 2/103 | 1.9 | | NA | |
| 34 | 16/383 | | 4.2 | | 104/1395 | | 7.5 | | 0.378 | | 33/621 | 5.3 | | 69/1005 | 6.9 | 0.097 | | | 6/119 | 5 | 21/426 | | 4.9 | 0.485 | | | 0/27 | 0 | 3/103 | 2.9 | | 0.175 | |
| 35B | 11/383 | | 2.9 | | 52/1395 | | 3.7 | | 0.809 | | 10/621 | 1.6 | | 15/1005 | 1.5 | 0.565 | | | 5/119 | 4.2 | 8/426 | | 1.9 | 0.316 | | | 1/27 | 3.7 | 4/103 | 3.9 | | 0.275 | |
| Distribution of serotypes among pneumococcal isolates (excluding NT isolates). P-value from chi-square test adjusted for village-level clustering. | | | | | | | | | | | | | | | | | | | | | | | | | | | | | | | | |  |
|  | | | | | | | | | | | | | | | | |  | | | | | | | | | | | | | | | |  |
